# Routine resite of peripheral intravenous devices every 3 days did not reduce complications compared with clinically indicated resite: a randomised controlled trial

**DOI:** 10.1186/1741-7015-8-53

**Published:** 2010-09-10

**Authors:** Claire M Rickard, Damhnat McCann, Jane Munnings, Matthew R McGrail

**Affiliations:** 1 Research Centre for Clinical and Community Practice Innovation, Griffith University, 170 Kessels Rd, Nathan Qld 4111, Australia; 2 School of Nursing and Midwifery, University of Tasmania, Newnham Drive, Launceston Tas 7250, Australia; 3 St Luke's Hospital, Calvary Tasmania, Launceston Tas 7250, Australia; 4 Gippsland Medical School, Monash University, Northways Rd, Churchill Vic 3842, Australia

## Abstract

**Background:**

Peripheral intravenous device (IVD) complications were traditionally thought to be reduced by limiting dwell time. Current recommendations are to resite IVDs by 96 hours with the exception of children and patients with poor veins. Recent evidence suggests routine resite is unnecessary, at least if devices are inserted by a specialised IV team. The aim of this study was to compare the impact of peripheral IVD 'routine resite' with 'removal on clinical indication' on IVD complications in a general hospital without an IV team.

**Methods:**

A randomised, controlled trial was conducted in a regional teaching hospital. After ethics approval, 362 patients (603 IVDs) were randomised to have IVDs replaced on clinical indication (185 patients) or routine change every 3 days (177 patients). IVDs were inserted and managed by the general hospital medical and nursing staff; there was no IV team. The primary endpoint was a composite of IVD complications: phlebitis, infiltration, occlusion, accidental removal, local infection, and device-related bloodstream infection.

**Results:**

IVD complication rates were 68 per 1,000 IVD days (clinically indicated) and 66 per 1,000 IVD days (routine replacement) (*P *= 0.86; HR 1.03; 95% CI, 0.74-1.43). Time to first complication per patient did not differ between groups (KM with log rank, *P *= 0.53). There were no local infections or IVD-related bloodstream infections in either group. IV therapy duration did not differ between groups (*P *= 0.22), but more (*P *= 0.004) IVDs were placed per patient in the routine replacement (mean, 1.8) than the clinical indication group (mean, 1.5), with significantly higher hospital costs per patient (*P *< 0.001).

**Conclusions:**

Resite on clinical indication would allow one in two patients to have a single cannula per course of IV treatment, as opposed to one in five patients managed with routine resite; overall complication rates appear similar. Clinically indicated resite would achieve savings in equipment, staff time and patient discomfort. There is growing evidence to support the extended use of peripheral IVDs with removal only on clinical indication.

**Registration number:**

Australian New Zealand Clinical Trials Registry (ANZCTR) Number ACTRN12608000421336.

## Background

Peripheral intravenous device (IVD) insertion is the most commonly performed invasive procedure in hospitalised patients, with an estimated 150 million peripheral intravenous devices placed each year in North America alone [1]. IVDs are vital for delivery of hydration, medicines and nutrition but are not without complications. Serious adverse outcomes are fortunately rare, with IVD-related bloodstream infection reported in a recent meta-analysis of 110 studies to occur in 0.1% of devices and 0.5 per 1,000 device days [2]. IVD treatment is more frequently interrupted by phlebitis, an irritation of the vein characterised by pain, tenderness on palpation, erythema, warmth, swelling, induration or palpable cord (thrombosis) of the cannulated vein; diagnostic algorithms usually require two or more of these conditions [3-5]. Phlebitis is in almost all cases a biochemical reaction to the mechanical irritation by the presence of the IVD and associated infusate [3], although phlebitis symptoms such as erythema may be misperceived as indicative of an infection. In fact, there is not a high correlation between phlebitis and device infection, and the Centers for Disease Control (CDC) states that infection is rarely associated with peripheral, as opposed to central, venous devices [3,6,7]. Fluid infiltration or 'tissuing' of devices is another common IVD complication which may also reflect the inflammatory (phlebitic) response of the vein, rather than simple misplacement of the device tip [8].

Early cohort studies noted an association between increased device time *in situ *and phlebitis [9,10]. This association was responded to with policies for routine device removal. Recommended timelines for routine resite have been extended over the past three decades from 24, to 48, then to 72 hours. Currently, 72- to 96-hour resite is recommended to reduce phlebitis by the CDC's 2002 Guidelines for the Prevention of Intravascular Device Infection, with the exemption that this is not required in children or those with poor veins [7]. Such policies cause increased workload in hospitals, where the task of removing and replacing well-functioning IVDs generally falls to busy nursing and junior medical staff. In addition, few patients welcome the prospect of additional venipuncture.

Despite the general clinical acceptance of routine IVD replacement as a phlebitis and infection prevention measure, it has not been supported by recent data. It may be that the risk of complications during the entire IVD treatment episode is similar, regardless of whether multiple short-dwell or fewer longer-dwell IVDs are used over this time. Three small (n = 47-60) randomised, controlled trials (RCTs) suggested routine replacement at 12, 24 or 48 hours may reduce phlebitis compared to resite on clinical indication, although a systematic review for the Swedish Council on Technology Assessment in Healthcare assessed these as low- to medium-quality studies providing 'limited scientific evidence' [11-14]. More recently, two well-conducted RCTs found no evidence of effect when comparing IVD replacement every 3 days with replacement only on clinical indication for medical and surgical inpatients [15,16]. The largest of these studies reported findings from 755 general medical and surgical patients with 1,428 IVDs and found a 5% difference in combined phlebitis and infiltration rates per patient (38% clinically indicated resite, 33% routine resite), suggesting a potential small clinical benefit of 3-day resite [15]. However, this difference was not statistically significant (RR 1.15; 95% CI, 0.95-1.40) and disappeared when overall cannulation time was considered (59.8/1,000 IVD days clinically indicated resite, 60.9/1,000 IVD days routine resite; RR 0.98; 95% CI 0.78-1.24) [15]. In addition, no clinically important or statistically significant differences were observed in the secondary endpoints of phlebitis, infiltration, occlusion, local infection or suspected bloodstream infection rates between study groups [15]. Another recent RCT in the 'hospital in the home' community setting also found no important clinical or statistically significant difference in phlebitis, occlusion or bloodstream infection rates in 316 patients when resite every 3 days was compared with clinically indicated resite [17]. A 2010 Cochrane Collaboration review concluded there was 'no conclusive evidence of benefit' of routine IVD resite and suggested organisations could consider adopting a resite on clinical indication policy [18]. There is growing evidence that routine IVD replacement may be ineffective, although caution has been urged in light of the large number (74% in both groups in the largest study to date) of reported devices inserted by a specialised IV team, a factor known to reduce complications [19].

Device insertion (and reinsertion) is unpleasant for patients, requires skilled and available clinical staff, and has associated costs for the health sector. If replacement only on clinical indication is safe and effective, this would have important benefits for patients and the health system. We report a RCT of 3-day routine IVD resite versus clinically indicated replacement in a medical-surgical hospital where IVDs were inserted by the general medical and nursing staff; the insitution did not have a specialised IV service.

## Methods

### Aim

The aim of the study was to compare the impact of 3-day routine resite, with clinically indicated resite, on peripheral IVD complications.

### Design

Open (nonblinded), parallel group RCT.

### Ethics

The study was approved by the Tasmanian State Human Research Ethics Committee. Written informed consent was obtained prospectively from all participants.

### Setting and sample

The study was undertaken at a large regional teaching hospital in Australia which functions as the tertiary referral centre for the northern half of the State of Tasmania. The hospital has more than 32,000 separations per annum, with a spectrum of medical and surgical specialties. Eligible patients were at least 18 years of age and scheduled or expected to have a peripheral IVD indwelling for at least 4 days, and they gave written informed consent. Exclusion criteria were immunosuppression, current bloodstream infection or an IVD already in situ for >48 hours. IVDs were inserted and cared for by the general nursing and medical staff; there was no special IV team or service.

### Sample Size

Sample size calculations were performed using PASS 2008 (Version 8.0.8; Kaysville, UT) to detect a change in rates by 30% (from 36% to 25%, two-tailed α = 0.05, 90% power) on the basis of the complication rates of routinely resited IVs in a previous study [16]. Although this indicated that n = 378 per group (total 756) were required, the study was ceased early (total n = 606 IVs) because all investigators left the employment of the institution. Consequently, study power was reduced, but remained over 80% (required minimum n = 282 per group).

### Recruitment

All adult patients admitted to the inpatient acute medical and surgical wards of the study hospital were screened by a full-time research nurse. This excluded paediatric, day-surgery, mental health, obstetric, critical care and dialysis units.

### Study procedures

Patients were randomly assigned (computer generated) in a 1:1 allocation ratio to either the 'routine replacement' (control) or 'clinically indicated replacement' (intervention) group. Assignment was concealed until randomisation by use of a telephone service. A tag was placed on the insertion site indicating the study group. All devices for the patient were managed as per randomised group. The intervention group did not have their IVD resited unless clinically indicated. This decision was made by the treating clinician (not the investigators), who ordered IVD resite if the device failed or phlebitis occurred and ongoing IV treatment was required. The control group had a new device relocated to a different site by the general medical or nursing staff every 3 days. Control devices could also be removed at any stage by the clinical staff if they were not required or if complications occurred. Clinical nursing and medical staff undertook insertion and follow-up care of all IVDs as per the CDC Guidelines [7].

Laboratory staff undertaking microbiological culture assessments were blinded to the study group. Due to the nature of the intervention, patients, research, and clinical staff were unable to be blinded. However, the investigators had no involvement in assessing or documenting complications.

IVDs were assessed by the clinical nursing staff on each nursing shift for complications as part of standard clinical practice in the hospital. Times and dates of device insertion and removal were recorded along with the reason for device removal and any protocol deviations. A full-time research nurse collected data from the hospital chart and sought clarification from patients and clinical staff if necessary. Microbiological investigations (device tip, blood cultures and site cultures) were performed by the clinical staff on clinical suspicion of infection by the treating clinician. Demographic and clinical data were collected on age, sex, diagnosis at hospital admission, phlebitis risk based on Tagar *et al*.'s classification (low/medium/high risk) [20], past history of phlebitis, any comorbidities requiring active medical treatment (e.g., type 2 diabetes or congestive heart failure), haemoglobin, concurrent infection at other sites, antibiotic therapy, type of surgery, type of infusate and any additives (and their level of irritability), vein and skin quality assessment, size of device, insertion site, health professional inserting the device, and setting for insertion, presence of other vascular devices, wound drains and urinary catheters. Vein quality was assessed as good (vein easy to visualise and easy to palpate with tourniquet on), fair (not easily visible but can palpate with tourniquet), or poor (veins small, scarred or difficult to palpate with tourniquet; may require heat pack to aid vasodilation). Skin quality was assessed as good (healthy, well hydrated, elastic), fair (mildly dehydrated, reduced elasticity), or poor (papery, dehydrated, or reduced elasticity).

### Analytic Approach

The primary outcome was a composite measure of any complication causing unplanned cannula removal prior to completion of IV treatment. The composite included phlebitis, infiltration, occlusion, accidental removal, local infection, and IV device-related bloodstream infection (IVD-BSI). These were also analysed individually as secondary endpoints. A composite measure was chosen due to the low rates of these conditions individually and to the assumption that they are comparable measures of 'infusion failure'; that is, the device can no longer be used to deliver treatment. This approach has been used in previous studies on the topic [15-17]. Phlebitis was defined as two or more of pain, erythema, purulence, streak formation, or a palpable venous cord [3]. Local infection IVD-BSI (bacteremia/fungemia with at least one positive blood culture obtained from a peripheral vein, clinical manifestations of infection, and no apparent source for the bloodstream infection (BSI) except the device with or without positive tip or entry site swab culture) were defined using CDC criteria [7]. Other secondary outcomes were time *in situ *(hours of catheterisation from insertion to removal, both per patient and per device) [7]; IVDs per patient (number of peripheral devices inserted to complete the course of treatment) [7]; costs (calculations based on 20 minutes nursing or medical insertion time at relevant rates [15], plus the cost of the required equipment (cannula, insertion pack including dressing and solution, gloves, saline, syringe, extension tubing and starter pack for all plus fluid bag, tubing and secondary tubing for medication administration for those patients requiring this) for insertions, nursing time and equipment to routinely remove IVDs that were otherwise functional, and the costs of treating any complications that occurred (e.g., IVD-BSI). Cost calculations were undertaken from the viewpoint of the hospital using negotiated wage costs and purchasing agreements for government hospitals in the State of Tasmania. Costs would be similar for other Australian hospitals.

All randomised patients were analysed by intention to treat. Each patient was randomised once and could have multiple IVDs, with subsequent IVD resites managed as per the randomised group. Relative incidence complication rates per 1,000 IVD days and 95% confidence intervals were calculated to summarise the impact of clinically indicated replacement relative to 3-day replacement. Kaplan-Meier survival curves were drawn to compare time to first IVD complication between patients in the two study groups. To assess for any potential impact of protocol violations, a per protocol analysis was also undertaken. All clinical and demographic variables were subjected to univariate testing against the primary endpoint to guide selection of possible covariates for the multivariable model. Cox proportional hazards regression modelling was used to examine the impact of age, gender, oncology status, number of comorbidities (nil, one, two, or more than two), IV gauge, site, vein quality, skin quality, oral antibiotics, IV antibiotics, wound drain, inserter occupation, initial versus subsequent IVDs, phlebitis in a preceeding IVD, haemoglobin level, parenteral nutrition, continuous versus intermittent infusion, patient risk category and study group on the outcome of time to complication events using an additive model [3,5,7,20-25]. In addition, to adjust for any inherent correlations or codependencies in the failure times of IVDs (i.e., same patient multiple failure-time data) within the Cox model, we also used the Prentice-Williams-Peterson conditional risk-set method [26]. The Mann-Whitney test was used to compare various secondary outcomes between study groups. Cost differences were calculated using arithmetic means and the *t*-test [27]. *P *values <0.05 were considered significant. All statistical data were entered and analysed using SPSS (Version 15.0; Chicago, IL) and Stata (Version 8.2; College Station, TX).

## Results

### Sample

**Figure 1:**
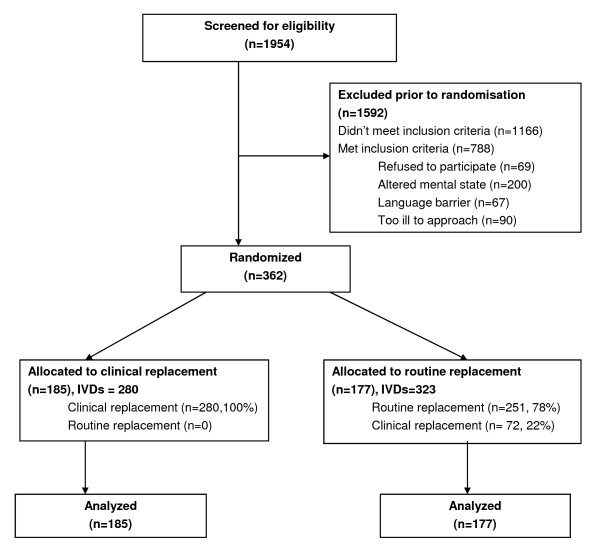
**Participant flowchart**.

Over a 10-month period, 1,954 patients were screened for eligibility. Of these, 788 were eligible, with 362 (46%) recruited into the study. The most frequent exclusion criterion was altered mental state that precluded consideration of consent as assessed by the research nurse. Altered mental state was generally related to older medical patients and the immediate postoperative phase for surgical patients. Reasons for exclusion are shown in Figure 1. The 362 patients were randomised into either the routine change group (n = 177 participants, 323 devices) or the clinically indicated replacement group (n = 185 participants, 280 devices). In total 50,173 IVD hours were studied (routine change group 23,288 hours, clinically indicated group 26,885 hours). More patients in the routine change group had an active infection (53% vs. 44%) and were receiving IV antibiotics (73% vs. 64%). However, as shown in Tables 1 and 2, the two groups were generally comparable at baseline for patient- and cannula-related factors.

**Table 1:** Baseline characteristics of study participants

Variable	3-Day Routine Change Group (n = 177)	Clinically Indicated Change Group (n = 185)
Sex - Male, n (%)	96 (54%)	103 (56%)
Age, mean (SD)	65.1 (17.3)	62.7 (15.4)
Reason for admission, n (%)		
• Gastrointestinal	46 (26%)	52 (28%)
• Respiratory	37 (21%)	34 (18%)
• Oncology	21 (12%)	21 (11%)
• Orthopaedic	16 (9%)	22 (12%)
• Cardiac	10 (6%)	10 (5%)
• Neurological	9 (5%)	10 (5%)
• Vascular	7 (4%)	7 (4%)
• Renal	16 (9%)	9 (5%)
• Other	15 (8%)	20 (11%)
Number of comorbidities, n (%)		
• None	6 (3.4%)	10 (5.4%)
• 1	22 (12.4%)	21 (11.4%)
• 2	44 (24.9%)	54 (29.2%)
• >2	105 (59.3%)	100 (54.1%)
Type of surgery, n (%)		
• Nil	124 (70%)	132 (71%)
• Gastrointestinal	22 (12%)	22 (12%)
• Orthopaedic	8 (5%)	15 (8%)
• Other	23 (13%)	16 (9%)
Most recent Hb - Mean (SD)	124.8 (23.2)	126.2 (21.9)

**Table 2:** Baseline infusion-related characteristics of study devices

Variable	**3-Day Routine Change Group (n = 323)**, n (%)	**Clinically Indicated Change Group (n = 280)**, n (%)
IV cannula gauge		
• 22	43 (13%)	46 (16%)
• 20	241 (75%)	201 (72%)
• 18	27 (12%)	30 (11%)
• Other	2 (1%)	3 (1%)
Vein assessment		
• Good	143 (44.3%)	107 (38.2%)
• Fair	160 (49.5%)	144 (51.4%)
• Poor	20 (6.2%)	29 (10.4%)
Skin integrity		
• Good	138 (43%)	104 (37%)
• Fair	178 (55%)	164 (59%)
• Poor	7 (2%)	12 (4%)
Past history of phlebitis	2 (0.6%)	4 (1.4%)
Insertion site		
• Hand	206 (64%)	187 (67%)
• Forearm	59 (18%)	49 (18%)
• Cubital fossa	51 (16%)	38 (14%)
• Other	7 (2%)	6 (2%)
Receiving infusate	241 (75%)	215 (77%)
pH of infusate - Mean (SD)	6.0 (0.5)	6.0 (0.4)
Receiving oral antibiotics	24 (7%)	24 (9%)
Receiving IV antibiotics	236 (73%)	176 (63%)
pH of IV antibiotics - Mean (SD)	6.9 (1.1)	6.9 (1.3)
Receiving other IV meds	190 (59%)	179 (64%)
pH of other IV meds - Mean (SD)	5.6 (2.6)	6.0 (2.8)
Wound drain	50 (16%)	40 (14%)
Urinary catheter	55 (17%)	41 (15%)
Other vascular device	26 (8%)	24 (9%)
Inserted by		
• Junior doctor	232 (72%)	207 (74%)
• Registered Nurse	67 (21%)	57 (20%)
• Senior doctor	24 (7%)	16 (6%)
Where inserted		
• Ward	216 (67%)	188 (67%)
• Emergency department	74 (23%)	70 (25%)
• Other	33 (10%)	22 (8%)
Current infection (site)		
• Respiratory	97 (30%)	49 (18%)
• Urinary	24 (7%)	19 (7%)
• Wound	16 (5%)	15 (5%)
• Other	29 (9%)	37 (13%)
• >1 site	5 (2%)	4 (1%)
N/A (none)	152 (47%)	156 (56%)
Risk of phlebitis (Tagar scale)		
• Low	174 (54%)	144 (51%)
• Medium	149 (46%)	136 (49%)
• High	0	0

### Effect of intervention on primary outcome

**Figure 2:**
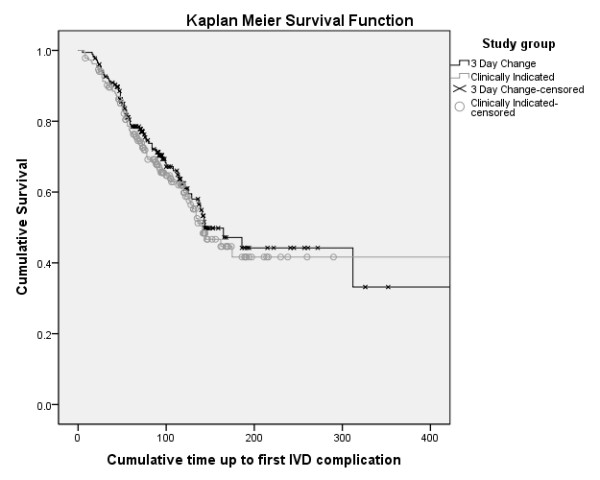
**Kaplan-Meier survival curve of time to first intravenous device complication per patient (log rank, *P *= 0.53)**.

Outcome data were available for all patients. Table 3 shows the rates of primary and secondary outcomes. Differences in complication rates between groups were not significantly different (routine replacement 66.0 per 1,000 IVD days; clinical replacement 67.8 per 1,000 IVD days; HR 1.03; 95% CI, 0.74-1.43; *P *= 0.86). As shown in Figure 2, the time to first complication per patient was also not significantly different between groups (Kaplan Meier [KM] with log rank *P *= 0.53). On crude rate per IVD, the catheters replaced on clinical indication had higher complication rates (110/280 or 39% vs. 91/323 or 28%; *P *= 0.004). However, total complication rates per patient (to deliver the course of IV therapy) were not significantly different (*P *= 0.39) between clinically indicated (76/185, 41%) and routine resite patients (64/177, 36%).

**Table 3:** Effect of intervention on primary and secondary endpoints

Outcomes	3-Day Routine Change Group (n = 177)	Clinically Indicated Change Group (n = 185)	**RR (95% CI)**, *P *Value
Primary:			
IVD complications per patient, n (%)	64 (36%)	76 (41%)	RR 1.14 (0.88, 1.47), p = 0.39
IVD complications per 1000 IVD days	66.0 (95% CI 49.8, 82.1)	67.8 (95% CI 52.6, 83.1)	HR 1.03 (0.74, 1.43), p = 0.86
Secondary:			
Phlebitis, n (%)	12 (7%)	18 (10%)	RR 1.44 (0.71, 2.89), p = 0.34
Infiltration, n (%)	53 (30%)	61 (33%)	RR 1.10 (0.81, 1.49), p = 0.57
Occlusion, n (%)	5 (3%)	4 (2%)	RR 0.77 (0.21, 2.80), p = 0.75
Accidental removal, n (%)	11 (6%)	16 (9%)	RR 1.39 (0.66, 2.92), p = 0.43
Local infection	0	0	-
IVD-related BSI, n (%)	0	0	-
IVD costs per patient, AUD$ mean (SD)	$55.42 ($35.26)	$43.35 ($26.78)	Mean difference $12.07 (95%CI $5.57, $18.56), p < 0.001

Patient- and device-related variables considered in the multivariable model were older age, number of comorbidities (nil, one, two or more than two), smaller cannula size, poor skin or vein integrity, IV antibiotics, insertion by medical staff and study group. None of these were found to be statistically significant. The final Cox proportional hazards model after adjusting for time found study group was not a significant factor (HR 1.02; 95% CI, 0.77-1.36; *P *= 0.89). Variance-adjustment testing for potential multiple-failures per patient (cannula data) found no inconsistency in significant results compared to the main Cox model.

### Protocol compliance

Compliance with the study intervention was achieved in 78% (251 of 323) of routinely replaced IVDs removed at Day 3 and 100% (280 of 280) of IVDs resited on clinical indication. Noncompliance in the routine resite group was generally related to high staff workloads on that day or to staff's perception that the patient was soon to be discharged or have therapy completed, and so the IVD remained *in situ *beyond Day 3. A per protocol analysis was performed including only those patients in the routine replacement group whose IVDs were all actually removed by 96 hours (n = 251). This found no significant difference (KM with log rank *P *= 0.16) in the rate of complications between groups (routine replacement 92 per 1,000 IVD days vs. clinically indicated 68 per 1,000 IVD days).

### Effect of intervention on secondary outcomes

There was no statistically significant difference in group outcomes for any phlebitis (*P *= 0.34), infiltration (*P *= 0.57), occlusion (*P *= 0.75), or accidental removal (*P *= 0.43). No cases of local infection or IVD-related bloodstream infection occurred in either group.

For overall IVD therapy (total cannulation time per patient for all devices), clinically indicated devices had a median therapy of 120 hours (quartiles 86.5 and 172.5 hours), and those replaced routinely had median therapy of 113 hours (quartiles 72 and 172 hours) (*P *= 0.21). For individual IVD dwell times, the clinically indicated group devices had a median dwell time of 85 hours (quartiles 51 and 121 hours), and those replaced routinely had a median dwell time of 71 hours (quartiles 48 and 90 hours) (*P *< 0.001). The maximum IVD dwell time was 1,023 hours (43 days) in the clinical replacement group, and this cannula was still working well on removal for completion of therapy.

The overall number of IVDs per patient was significantly less (*P *= 0.004) for those replaced on clinical indication (mean 1.5, SD 0.8, median 1, quartiles 1 and 2) than for those routinely replaced (mean 1.8, SD 1.1, median 1, quartiles 1 and 2). A total of 22% of patients in the routinely replaced group had three or more IVDs compared with 9% in the clinical indication group. A total of 82 (28%) IVDs in the routine replacement group were resited after 3 days despite functioning well and ongoing treatment being required. (The remainder removed at this time were no longer required and so not resited, or infusion failure had already occurred.) Mean hospital costs per patient for the course of IV therapy were significantly higher (*P *< 0.001) for those managed with routine resite (mean $55.42, SD $35.26) compared with resite on clinical indication (mean $43.35, SD $26.78).

## Discussion

The finding that 3-day routine resite was not an effective intervention was consistent across the intention-to-treat primary analysis and the per protocol analysis. There remained no effect when events were expressed per patient or per 1,000 IVD days. Neither composite nor individual complication rates differed between groups, and there were no cases of local or device-related bloodstream infection. It appears safe and practical to leave IVDs *in situ *as long as they are functioning well and are still needed for clinical treatments.

All IVDs will fail eventually, but this study shows that artificially shortening the lifespan of individual catheters does not reduce the overall complication rates over the course of therapy. Our results indicate that the average duration of IV therapy is 5-6 days and that many catheters can remain complication-free for this period. If catheters are not routinely resited, the median dwell time would remain within the 72-96 hours recommended by the CDC, but about 10% would remain *in situ *for longer (in this study up to 43 days with no complications). Our data show that a policy of resite on clinical indication would mean that one of every two patients would need a single cannula to receive treatment, whereas a 3-day change policy would result in only one in five patients having this scenario, with the rest requiring multiple cannulations and therefore additional pain and inconvenience.

The results are consistent with the findings of recent RCTs in both hospitals and the community that have found no benefit in routinely resiting IVDs every 3 days [15-17]. In these studies, many cannulae were inserted by an expert IVD team [15-17], which may have minimised complications in both groups [19]. Our study confirms and extends these findings into the general medical/surgical setting without an IV team where IVDs were inserted by a variety of nursing and medical staff. Data from this study were included in a 2010 Cochrane Collaboration systematic review and meta-analysis on the topic [18]. This review included six trials (n = 3,455) and reported no clinically important or statistically significant difference in catheter-related bloodstream infection or phlebitis between IVDs that were routinely resited (at 48-96 hours) or resited on clinical indication, yet there were significantly lower costs in the group resited on clinical indication [18].

The belief that routine resite of IVDs will prevent complications appears to stem from early observational studies that noted longer-dwelling IVDs had more complications than shorter-dwelling IVDs [9,10]. This is intuitively true, given, for example, that an IVD *in situ *for 6 days has twice the time exposure of risk than an IVD *in situ *for 3 days. However, this does not prove that two sequentially inserted IVDs (in the same patient), both used for 3 days, have a combined lower risk over time than the 6-day dwell device. Indeed, this and other recent trials strongly suggest that the risk for the patient over the 6-day period is similar. Well-designed cohort studies with modern catheter materials suggest that the daily risk of phlebitis is relatively stable after the first 24 or 48 hours [3,21,28-31]. The peak in phlebitis between 24 and 48 hours is likely associated with the time taken by the body to mount a biological response after the instigation of therapy; those most likely to develop phlebitis will do so at this time.

The results support the extension of the use of peripheral IVDs beyond the 72-96 hours currently recommended by the CDC [7]. There is incongruity in the CDC recommendations; they recommend *not *to routinely resite IVDs in children or in those with limited venous access. If it is safe in these populations, it is unclear why it would be necessary to routinely resite adults or those with better veins. Higher-risk cannulae such as central venous devices are no longer recommended by the CDC for routine replacement, because trials showed this was not of benefit [7]. Our study also confirms that the CDC guidelines are not always complied with; one fifth of IVDs in the routine change group were not replaced by this time. However, the per protocol analysis showed that the intervention remained ineffective even for those who truly had their IVDs resited every 3 days.

Limitations of the study included a 9% higher frequency of IV antibiotics and concurrent infection in the routine resite group. This may have put the group at higher risk due to vein irritation, or conversely it protected against bacterial entry. Neither variable was significant in the multivariable model. The unblinded study design was unavoidable, but also a limitation. Our use of clear outcome measures, a full-time research nurse and laboratory staff blinded to culture assessments should have reduced the risk for potential bias. Resource constraints prematurely ended recruitment, thus reducing the anticipated power of the study from 90% to 80%.

Routine IVD resite involves pain for patients, staff procedural time, equipment costs and environmental waste. Contemporary evidence suggests the current policy for routine resite by 72-96 hours is ineffective and should be replaced with a 'resite on clinical indication' policy. It remains imperative that clinical staff monitor IVDs closely and that a daily review of the need for continuing therapy be made, with cessation as soon as possible; the only no-risk IVD is no IVD. Of the 4.3 million acute hospital admissions in Australia each year (excluding day cases), over half have IV treatment [15,32]. Conservatively, if even 2 million courses of IV therapy were managed with clinically indicated rather than routine resite, this would save the unnecessary insertion of approximately 660,000 IVDs and free 280,000 hours of staff insertion time. Assuming our costs are similar to those in other Australian hospitals, a change to resite on clinical indication would save approximately AUD$24 million nationally each year.

## Conclusions

Although larger, multisite trials are required, evidence to date suggests that routine resite of peripheral IVDs increases patient discomfort and healthcare costs, but does not reduce IVD complications as has traditionally been thought.

## Competing interests

The authors declare that they have no competing interests.

## Authors' contributions

CMR designed the study, applied for funding and drafted the manuscript. DM participated in study design and manuscript preparation, and managed the study. JM recruited patients, ensured protocol integrity and collected data. MRM undertook statistical analyses and assisted with drafting the manuscript. All authors read and approved the final manuscript.

## Authors' information

CMR, PhD is Professor, Research Centre for Clinical and Community Practice Innovation, Griffith University, Australia. DM holds a Masters by Research and is Senior Lecturer, University of Tasmania, Australia. JM is an RN and Nurse Unit Manager, Calvary Tasmania, Australia. MRM is a PhD qualified Biostatistician, Gippsland Medical School, Monash University, Australia.

## Pre-publication history

The pre-publication history for this paper can be accessed here:

http://www.biomedcentral.com/1741-7015/8/53/prepub
